# *Volvariella volvacea* Polypeptide Mitigates Alcohol-Induced Liver Injury: A Multi-Omics Study

**DOI:** 10.3390/foods14091557

**Published:** 2025-04-29

**Authors:** Bingzhi Chen, Juanqin Chen, Huihua Wu, Fangyi Zhang, Lili Chen, Weibin Zhang, Jing Yang, Li Yuan, Yuji Jiang, Youjin Deng

**Affiliations:** 1 College of Food Science, Fujian Agriculture and Forestry University, Fuzhou 350002, China; cbz_2006@163.com (B.C.); hihihaha22@163.com (J.C.); 15259307035@163.com (H.W.); zfy_7015@163.com (F.Z.); llchen04@163.com (L.C.); zwb20020201@163.com (W.Z.); 18703568695@163.com (J.Y.); 18798168382@163.com (L.Y.); 2 Mycological Research Center, Fujian Agriculture and Forestry University, Fuzhou 350002, China; 3 Key Laboratory of Subtropical Characteristic Fruits, Vegetables and Edible Fungi Processing (Co-Construction by Ministry and Province), Ministry of Agriculture and Rural Affairs, Fuzhou 350002, China

**Keywords:** straw mushroom, acute alcoholic hepatitis, transcriptomics, metabonomics, joint analysis, hepatoprotective mechanisms

## Abstract

This study investigated the hepatoprotective mechanisms of *Volvariella volvacea* fruiting body polypeptide (VVFP, 1–3 kDa) against acute alcohol-induced liver injury using multi-omics approaches. Male ICR mice pretreated with VVFP (100–400 mg/kg) showed significantly prolonged alcohol tolerance latency (*p* < 0.05) and accelerated sobriety recovery compared to controls. Integrated transcriptomics and metabolomics revealed VVFP’s dual regulatory effects: (1) transcriptional regulation of 36 endoplasmic reticulum stress genes (e.g., *ERP57*, *Derl*) through protein processing pathways (KEGG:04141), and (2) metabolic modulation of 23 hepatic metabolites, particularly phosphatidylcholines and organic acids, via amino acid biosynthesis and glycerophospholipid metabolism. Cross-omics analysis identified eight coregulated genes (*Got1*, *Arg2*, *Srm*, etc.) interacting with key metabolites (4-guanidinobutyric acid, GABA) through linoleic acid metabolism. These findings demonstrate VVFP’s therapeutic potential as a functional food ingredient by highlighting its ability to simultaneously target hepatic stress responses and metabolic homeostasis during alcohol detoxification.

## 1. Introduction

Bioactive peptides (typically 1–10 kDa) demonstrate multifunctional physiological activities through specific amino acid sequences and secondary structures [1]. Emerging evidence highlights their therapeutic potential in metabolic regulation, particularly in modulating hypertension via angiotensin converting enzyme inhibition [2], improving insulin sensitivity [3], and enhancing antioxidant defense systems [4]. Notably, recent studies emphasize the crucial interplay between dietary peptides and gut microbiota in mediating these health benefits [5].

*Volvariella volvacea* (Bull.) Singer, a thermophilic edible mushroom widely cultivated in Southeast Asia, is rich in protein (22.7% dry weight) and bioactive polysaccharides with demonstrated immunomodulatory effects (e.g., 58.4% HepG2 cell inhibition at 500 μg/mL) [6,7,8,9]. While its β-glucan-rich polysaccharides have been extensively studied for antitumor properties [10], proteomic investigations remain limited [11]. Our prior work isolated low-molecular-weight peptides of *Volvariella volvacea* fruiting body polypeptide (VVFP, 1–3 kDa) from enzymatic hydrolysates, revealing dose-dependent antioxidant capacity (2,2-Diphenyl-1-picrylhydrazyl scavenging rate: 78.2% at 2 mg/mL) and preliminary hepatoprotective effects in murine models (200 mg/kg VVFP reduced serum Alanine aminotransferase (ALT) by 41.7%) [12,13,14].

To bridge the current research gap in VVFP applications, this investigation employs an integrated transcriptomic–metabolomic approach to systematically elucidate the dose-dependent hepatoprotective mechanisms of VVFP. By synergizing behavioral assessments with multi-omics profiling (RNA-seq and UHPLC-MS/MS), we aim to establish a gene-metabolite regulatory network that deciphers the molecular basis of VVFP’s efficacy against alcohol-induced liver injury. This methodological framework pioneers the application of food omics in edible mushroom research, providing critical insights for translating traditional fungal resources into evidence-based functional food development.

## 2. Materials and Methods

### 2.1. Materials and Reagents

Male ICR mice (SPF grade, 25 ± 2 g, 3–4 weeks old) were obtained from Wu’s Experimental Animal Trade Co., Ltd. (Certificate No. SCXK 2023-0008, Fuzhou, China). Diphenyl diester dropping pills (Zhejiang Pharmaceutical Co., Ltd., Huzhou, China) and 56% (*v*/*v*) Hongxing Erguotou (Beijing Red Star Co., Ltd., Beijing, China) were used as the positive control and alcohol source, respectively. Key reagents included the following: E.Z.N.A.™ Plant RNA Kit (Omega Bio-tek, D2480, Norcross, GA, USA), TRIzol™ Reagent (Thermo Fisher, 15596026, Waltham, MA, USA), and HPLC-grade solvents (methanol: A452; acetonitrile: 51101, Thermo Fisher, Waltham, MA, USA). All chemicals were analytical-grade unless specified.

### 2.2. VVFP Extraction and Purification

Freeze-dried *V. volvacea* fruiting bodies were homogenized in deionized water (1:20 *w*/*v*) using ultrasonic disruption with 600 W, 20 kHz, 30 min (Scientz-IID, Ningbo, Zhejiang, China). Then, the sample was leached for 1 h, centrifuged at 6000 r/min for 15 min, and the supernatant was taken to adjust the pH to the isoelectric point. After 1 h of settling, it was centrifuged again at 6000 r/min for 15 min to yield the precipitate, which is *V. volvacea* protein. The *V. volvacea* protein solution, which had a material-to-liquid ratio of 1:50, was hydrolyzed by 7000 U/g alkaline protease at pH 11 and 45 °C for 2.5 h. The mixture was inactivated at 95 °C for 20 min, centrifuged for 20 min at 6000 r/min, and the supernatant was obtained. The VVFP was dialyzed and separated with 3 kD and 1 kD dialysis bags to obtain VVFP with a molecular weight of 1–3 kD, which was freeze-dried and stored in a drying dish for subsequent experiments. The total protein content of VVFP at this molecular weight (1–3 kD) was 71.12%, ash content was 12.75%, moisture content was 8.44%, and the remaining content was 7.69%. LC-MS/MS analysis showed that its molecular weight was mainly 1.5748 kD [13].

### 2.3. Animal Experiments

After a week of adaptive feeding, gastric perfusion was started for the 72 healthy male mice, which were randomly divided into 6 groups of 12 mice each: blank group (CK, 10 mL/kg), model group (MK, 10 mL/kg), positive control group (P, 200 mg/kg), low-dose polypeptide group (L, 100 mg/kg), medium-dose polypeptide group (M, 200 mg/kg), and high-dose polypeptide group (H, 400 mg/kg). The blank group received the same volume of normal saline, the positive control group received bidentate-dropping pills in deionized water, and the VVFP group received the same volume of VVFP solution according to different concentrations. Each mouse was weighed every 5 days and its dosage was modified based on its weight. Following the final dosage, every patient—aside from the blank group—was given 12 mL/kg Hongxing Erguotou by gavage every 5 h to create an acute ALD model. The modeling period lasted 12 h, and the patients underwent fasting without water. Following a cervical dislocation, the liver was promptly removed and stored in liquid nitrogen at −80 °C to determine the liver index later on. All these animal experiments were approved by the Animal Ethics Committee of Fujian Agriculture and Forestry University with the number FS-2023-048.

### 2.4. The Determination of Drunkenness and Sobering Time

After the gavage of Hongxing Erguotou, we started recording the amount of time the mice were inebriated and started to sober up. The precise manner of operation was as follows: Place the mice’s abdomens top-down on a horizontal table. If the mice maintain this position for longer than thirty seconds, their righting reflex will vanish and they will become inebriated. This should be recorded as the drunkenness time. The mouse has recovered its righting reflex and is no longer intoxicated if it can assume the belly-down position in less than thirty seconds. We refer to this period as sobering.

### 2.5. Liver Index Detection

During the experiment, the body weight was recorded every five days. After dissecting and taking out the liver, it was washed with normal saline until there was no blood, and the water was drained with filter paper to calculate the liver index:

Liver index = liver mass/mouse weight × 100%

### 2.6. Transcriptome Analysis

Using the TRIzol method (Invitrogen, Carlsbad, CA, USA), the total RNA from mouse liver tissue was extracted, and its concentration and OD value were determined. Beijing Ovison Company (Beijing, China) received high-quality liver tissue RNA (concentration >50 ng/µL and total sample amount >2 µg) and was tasked with supporting transcriptome sequencing. The transcriptome of the mouse liver tissue was sequenced using the Illumina Novaseq 6000 platform by the Beijing Allwegene Technology Company Limited (Beijing, China), and paired-end 150 bp reads were generated. Then, the amount of mRNA expressed in the liver was calculated with HTSeq (v0.5.4) with fragments per kilobase of transcript per million fragments mapped (FPKM) [15]. The differentially expressed genes (DEGs) were screened according to |log₂FoldChange| > 1 and FDR < 0.005. Then, these DEGs were subject to Gene Ontology (GO) functional annotation, and Kyoto Encyclopedia of Genes and Genomes (KEGG) pathway analysis [16].

### 2.7. Metabonomics Analysis

Weigh 25 mg of mouse liver tissue precisely, then add 500 μL of the extracted mixture (methanol:acetonitrile:water = 2:2:1), stir thoroughly, and grind for 4 min at 35 Hz using a Qualcomm tissue grinder. Finally, apply ultrasonic treatment for 5 min in an ice water bath. Let the sample stand in a refrigerator at −40 °C for one hour after completing the previous stages two or three times. Finally, the supernatant for detection after centrifuging at 12,000 r/min for 15 min at 4 °C. Using UHPLC-MS/MS technology, the metabonomics of the mouse liver tissue were identified, the metabolites that differed significantly between samples were eliminated, and the metabolites’ interactions were examined. Statistical analysis was performed by applying Orthogonal Partial Least Squares Discriminant Analysis (OPLS-DA). Based on the criteria of a *p* < 0.05, a Variable Importance in the Projection (VIP) value ≥ 1, and a fold change (FC) < 0.67 or >1.5, significant differentially expressed metabolites among different samples were screened, and their interactions were analyzed. Subsequently, Kyoto Encyclopedia of Genes and Genomes (KEGG) enrichment analysis was conducted on these differential metabolites.

### 2.8. Multi-Omics Joint Analysis

The relationship between genes and metabolites was discovered by a combined transcriptome and metabolomics investigation. We can learn more about the regulatory interaction between metabolites and differential genes related to alcoholic hepatitis by examining the regulatory network between the screened differential genes and metabolites.

### 2.9. Statistical Analysis of Data

Data processing was undertaken using SPSS Statistics 25.0, and visualization was carried out using GraphPad Prism 10 and the R software (4.5.0). To record the values, we used “mean standard deviation (x s)”. For significance analysis, the method of multiple comparisons was employed. A substantial difference between the data was present when *p* < 0.05, while an extremely significant difference between the data was present when *p* < 0.01.

## 3. Results

### 3.1. Ethanol Metabolism Modulation by VVFP

The therapeutic efficacy of VVFP was quantitatively assessed through intoxication latency (time to loss of righting reflex) and sobriety recovery duration. As shown in Table 1, VVFP administration demonstrated dose-dependent effects, with the medium-dose group (M-VVFP, 200 mg/kg) exhibiting optimal performance. Compared to the alcohol-induced model group (MK), M-VVFP prolonged intoxication latency 7.43-fold (MK: 9.33 ± 0.47 min, 95% Confidence Intervals (CI) [8.30, 10.36] vs. M: 69.33 ± 2.63 min, 95% CI [63.56, 75.10], *p* < 0.01) and reduced sobriety duration by 65.1% (MK: 216.67 ± 3.86 min, 95% CI [208.12, 225.22] vs. M: 75.67 ± 4.19 min, 95% CI [66.42, 84.92], *p* < 0.01).

### 3.2. Hepatoprotective Efficacy Assessment

The liver index was utilized as a key pathological indicator for assessing alcohol-induced hepatic edema. As demonstrated in Table 2, acute ethanol exposure (12 mL/kg) elevated this index by 26.3% versus controls (4.48% vs. 5.66%, *p* < 0.001), reflecting ethanol-induced hepatocellular damage and fluid retention. Compared to the model group (MK), the positive control (Group P) and VVFP-treated groups exhibited significant reductions in both liver mass and edema index (*p* < 0.01). Dose-dependent attenuation of hepatic edema was observed; the medium-dose group (200 mg/kg) restored the liver index to near-physiological levels (4.71 ± 0.09%, 95% CI [4.51, 4.91], a 16.8% reduction compared to the model). Notably, the high-dose group (400 mg/kg) achieved a 16.9% improvement (4.700 ± 0.075%, 95% CI [4.53, 4.87], *p* < 0.05), demonstrating a therapeutic plateau effect beyond 200 mg/kg. These findings establish VVFP’s dose-responsive hepatoprotective capacity, matching the efficacy of the positive control (bidentate pill) in alcohol-induced liver injury management.

### 3.3. Transcriptomic Profiling of Hepatic Responses

#### 3.3.1. Identification of Differentially Expressed Genes (DEGs)

**Figure 1:**
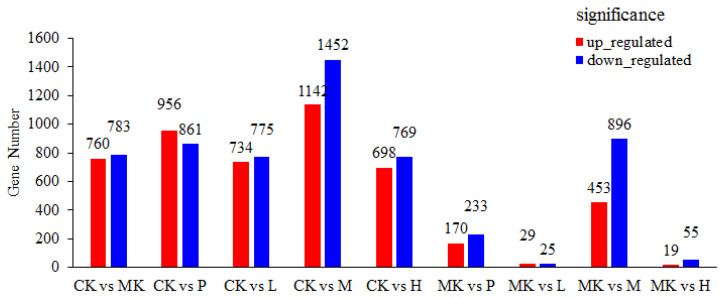
Statistical histogram of the number of differentially expressed genes.

RNA sequencing analysis of 18 liver samples (n = 3 per group) identified 10,810 DEGs, comprising 5849 down-regulated and 4961 up-regulated genes. Comparative analysis revealed distinct transcriptional responses across treatment groups (Figure 1). Specifically, the MK, P, L, M, and H groups exhibited 1543, 1817, 1509, 2594, and 1467 DEGs, respectively, compared to the CK group. When compared to the MK group, the P, L, M, and H groups showed 403, 54, 1349, and 74 significantly altered genes, respectively. The M-dose group (200 mg/kg VVFP) demonstrated the most pronounced transcriptomic alterations, exhibiting 7.6-fold more MK-responsive DEGs than other treatment groups (1349 vs. 54–403). This robust modulation of alcohol-induced gene expression patterns substantiated the selection of the M-dose for subsequent mechanistic investigations.

**Figure 2:**
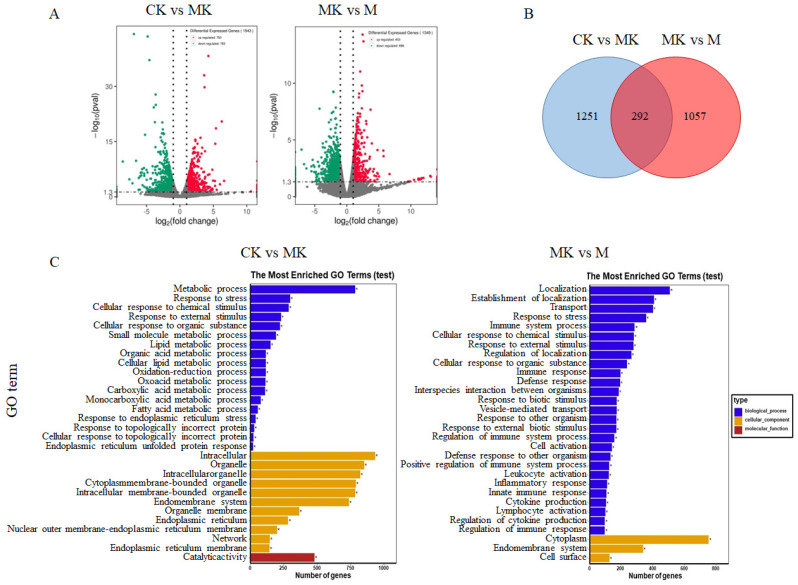
Analysis of differentially expressed genes among the CK, MK, and M groups. (**A**) Volcanic plot of differentially expressed genes; (**B**) Venn diagram of differentially expressed genes; (**C**) GO annotation plots of differentially expressed genes; * denotes significantly enriched GO term. Note: red dots, green dots, and gray dots represent significantly up-regulated, down-regulated, and non-significantly differentially expressed genes, respectively.

The volcano plot of differentially expressed genes between different groups can more intuitively reflect the distribution of differentially expressed genes between the two groups. As shown in Figure 2A, there are 1543 differentially expressed genes between the CK group and the MK group, indicating that oral administration of alcohol affects the gene expression level of normal experimental mice. After one month of oral administration of VVFP, as shown in Figure 1, there were 1349 differentially expressed genes between the MK group and the M group, which is significantly reduced compared with the CK group and the MK group, indicating that VVFP can reduce the number of differentially expressed genes caused by alcohol-induced liver damage. Venn diagram analysis (Figure 2B) delineated the unique and shared DEG patterns among experimental conditions. The CK vs. MK comparison revealed 1251 unique DEGs, while the MK vs. M group comparison identified 1057 distinctive DEGs. Notably, 292 DEGs were co-regulated in both comparisons, suggesting their potential role in mediating VVFP’s therapeutic effects.

#### 3.3.2. GO Function Annotation and Enrichment Analysis

Gene Ontology (GO) analysis, encompassing biological processes (BP), cellular components (CC), and molecular functions (MF), was performed to characterize the functional implications of transcriptomic alterations. As shown in Figure 2C, the metabolic process (342 up-regulated genes and 443 down-regulated genes) is the most abundant in BP among the differentially expressed genes in the CK and MK groups. Also notable are stress response (112 genes up-regulated and 187 genes down-regulated) and cellular response to chemical stress (120 genes up-regulated and 169 genes down-regulated). In CC, it is mainly intracellular (408 genes are up-regulated and 527 genes are down-regulated) and organelle (366 genes up-regulated and 487 genes down-regulated). There are many genes involved in intracellular organelle (350 up-regulated genes and 474 down-regulated genes). In terms of MF, it is mainly enriched in catalytic activity (243 genes up-regulated and 238 genes down-regulated).

As shown in Figure 2C, the differentially expressed genes between the MK group and M group are mainly concentrated in muscle localization (145 up-regulated genes and 365 down-regulated genes) in BP. Also notable are establishment of localization (128 genes up-regulated and 281 genes down-regulated), stress response (82 genes up-regulated and 277 genes down-regulated), and immune system process (30 genes up-regulated and 257 genes down-regulated). In CC, it is mainly in the cytoplasm (cytoplasm, with 271 up-regulated genes and 482 down-regulated genes), endomembrane system (141 genes up-regulated and 197 genes down-regulated), and enriched in cell surface (14 up-regulated genes and 112 down-regulated genes). There is no significant difference in genes in MF. The predominance of BP terms across both comparisons suggests that VVFP’s hepatoprotective mechanism primarily involves the modulation of biological processes rather than specific molecular functions or cellular components.

#### 3.3.3. Pathway Enrichment and Mechanistic Insights

**Figure 3:**
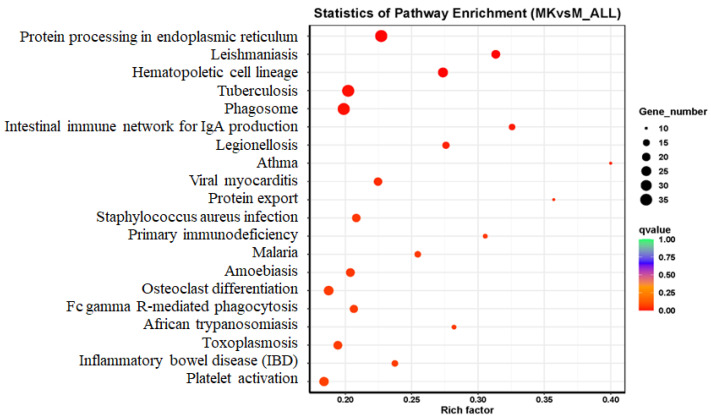
Bubble chart of KEGG enrichment for differentially expressed genes.

KEGG pathway analysis systematically delineated alcohol-induced hepatic perturbations and VVFP’s therapeutic targets. Rich factor, Qvalue, and Gene_number are used to measure the enrichment degree of KEGG. The greater the Rich factor value, the closer the Qvalue value is to zero, indicating a greater enrichment degree. Figure 3 shows the 20 pathways with the most significant enrichment of differential genes between the MK group and the M group. There are 3090 differential genes enriched to KEGG together, including 660 up-regulated genes and 2430 down-regulated genes. According to KEGG enrichment analysis, the differential genes were mainly concentrated in protein processing in the endoplasmic reticulum (36 up-regulated genes and 1 down-regulated gene), Hematopoietic cell lineage (0 up-regulated genes and 26 down-regulated genes), Tuberculosis (2 up-regulated genes and 34 down-regulated genes), Phagosome (3 up-regulated genes and 33 down-regulated genes), Osteoclast differentiation (1 up-regulated gene and 23 down-regulated genes), Toxoplasmosis (5 up-regulated genes and 16 down-regulated genes), and platelets.

**Figure 4:**
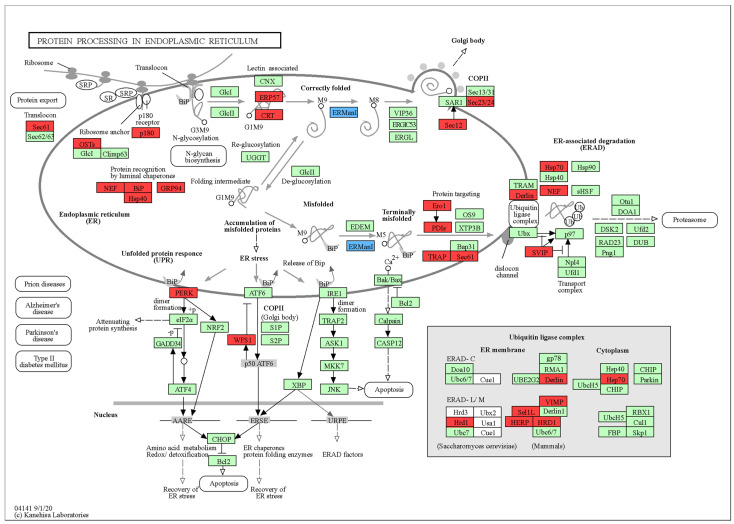
Protein processing in the endoplasmic reticulum pathway diagram between the MK group and M group. A red box indicates that the differential gene is significantly up-regulated; a blue box indicates that the differential gene is significantly down-regulated; a green box indicates that these genes were annotated during the transcriptome data annotation process.

The protein processing in the endoplasmic reticulum pathway (KEGG: 04141) between the MK group and the M group is shown in Figure 4, where blue genes are down-regulated and red genes are up-regulated. VVFP primarily influences the function of the endoplasmic reticulum in mouse hepatocytes in cases of acute alcohol-induced liver injury. It does this by up-regulating the expression of 36 differential genes, including *ERP57* (ENSMUSG00000027248 (1.5981-fold)), *Sec61* (ENSMUSG00000030082 (1.2862-fold), ENSMUSG00000053317 (1.0803-fold)), and *Derl* (ENSMUSG00000075701 (1.1543-fold)), and down-regulating the expression of *Man1* (ENSMUSG00000037306 (−1.2763-fold)) differential genes. VVFP up-regulated *ERP57* (a chaperone facilitating disulfide bond formation and glycoprotein folding), *Sec61* (a translocon restoring protein import efficiency), and *Derl* (an ERAD component promoting misfolded protein degradation), collectively alleviating ER stress by enhancing folding capacity, resolving translocation bottlenecks, and improving proteostasis. Additionally, down-regulation of *Man1* (a lectin involved in glycoprotein export) reduced ER retention of misfolded glycoproteins, further mitigating alcohol-induced ER dysfunction. This multi-target action highlights VVFP’s ability to restore ER homeostasis and protect hepatocytes. The findings support the hypothesis that VVFP modulates endoplasmic reticulum protein processing pathways, thereby attenuating alcohol-induced ER stress and hepatocyte damage [17,18,19]. These results align with prior studies demonstrating alcohol’s disruption of ER homeostasis [20,21], which triggers ER stress and cellular injury. Pharmacological interventions, such as VVFP, offer therapeutic potential by counteracting these pathological mechanisms.

### 3.4. Metabonomics Analysis of Mouse Liver

#### 3.4.1. Screening of Differential Metabolites

**Figure 5:**
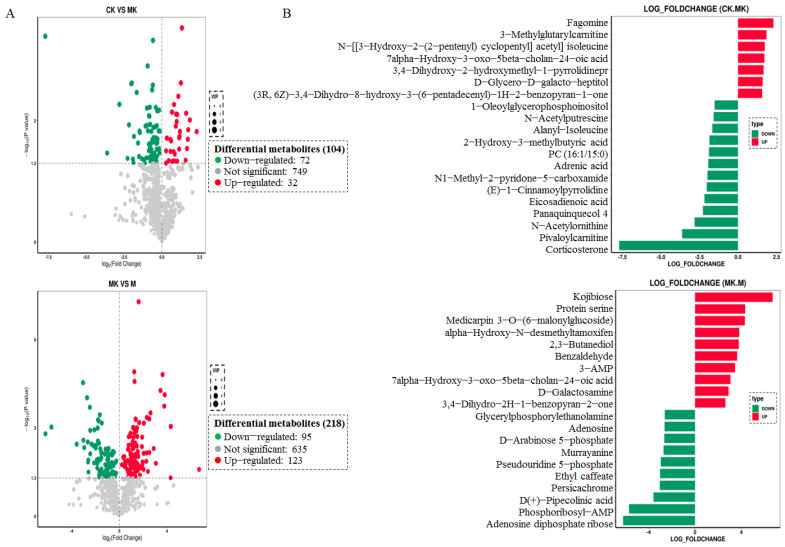
Metabolome analysis comparison. (**A**) Volcanic map of differential metabolites in different groups; (**B**) bar chart of differential metabolite multiples. Note: Red dots, green dots, and gray dots represent significantly up-regulated, down-regulated, and non-significantly differentially expressed genes, respectively.

The high dimensionality and high variability of metabolomics data require accurate identification of differential metabolites through multivariate statistical analysis combined with comprehensive evaluation. Volcano plots were generated based on the screening results, with a comparative analysis between groups presented in Figure 5A. In the CK vs. MK comparison, 104 differential metabolites were identified, including 32 significantly up-regulated and 72 significantly down-regulated species. Similarly, the MK vs. M comparison revealed 218 differential metabolites, comprising 123 up-regulated and 95 down-regulated compounds.

Figure 5B illustrates the top 20 differential metabolites showing the largest absolute logFC values in each comparison group. Fold changes of differential metabolites were visualized using histogram plots. In the CK vs. MK comparison, among the seven significantly up-regulated metabolites, fagomine and 3-methylglutarylcarnitine were notably enriched. Thirteen metabolites, including corticosterone, pivaloylcarnitine, and N-acetylornithine, predominated in the down-regulated metabolite cluster. Conversely, the MK vs. M comparison demonstrated 10 down-regulated metabolites (e.g., phosphoribosyl-AMP and ribose-AMP adenosine diphosphate) alongside 10 up-regulated compounds (such as protein serine and kojibiose). Most differential metabolites were associated with alcoholic liver injury. Notably, some metabolites, such as corticosterone (a stress hormone), may reflect systemic (non-hepatic) effects of alcohol toxicity. These effects include alcohol-induced intestinal epithelial permeability, gut microbiota dysbiosis, and systemic inflammation, which are exacerbated by corticosterone–alcohol interactions [22].

#### 3.4.2. KEGG Enrichment Analysis of Differential Metabolites

**Figure 6:**
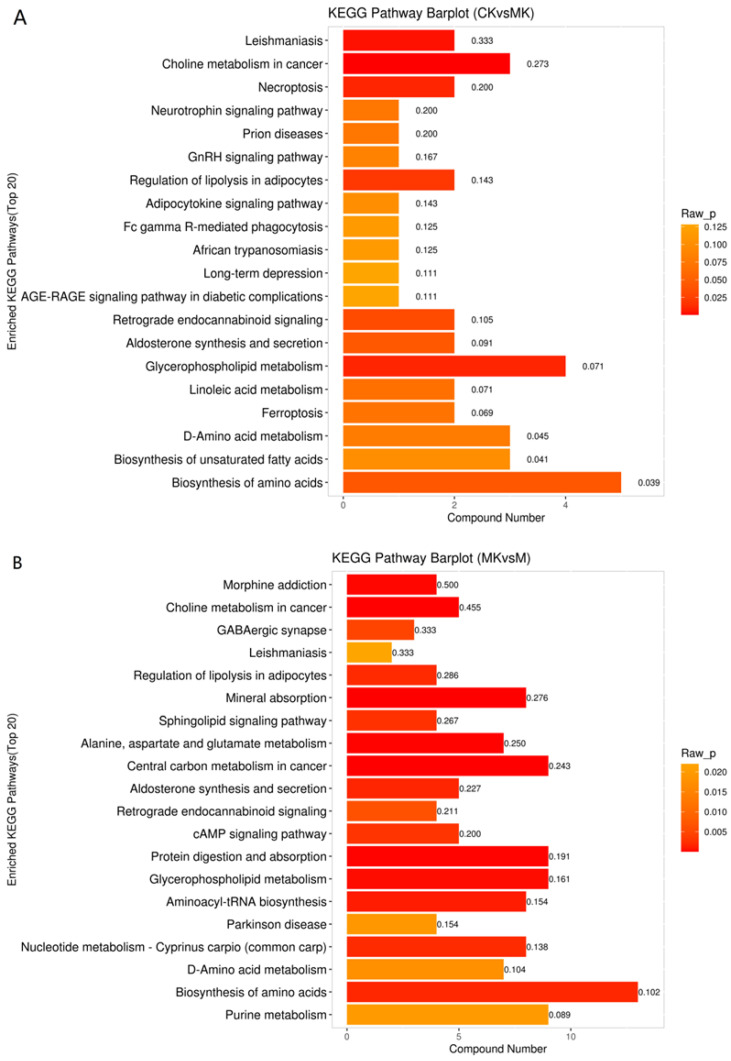
KEGG enrichment histogram of differential metabolites: (**A**) CK vs. MK; (**B**) MK vs. M. Note: Raw_P represents *p*-value.

To elucidate the hepatoprotective mechanism of VVFP against acute alcohol-induced liver injury, KEGG pathway enrichment analysis was performed to identify biologically relevant metabolic pathways. As shown in Figure 6A, the CK vs. MK comparison revealed significant enrichment in amino acid biosynthesis, glycerophospholipid metabolism, and choline metabolism. Correspondingly, Figure 6B demonstrates that the MK vs. M comparison exhibited enriched pathways including protein digestion/absorption, central carbon metabolism in cancer, and shared pathways with the former comparison (glycerophosphate metabolism and amino acid biosynthesis). Notably, these overlapping pathways were prioritized for investigating VVFP’s preventive mechanisms.

Consistent with established pathological mechanisms, hepatic injury induces systemic amino acid dysregulation. Following VVFP intervention, 13 differentially abundant metabolites were detected in amino acid biosynthesis pathways. Specifically, elevated concentrations of nine metabolites (e.g., L-proline, L-tryptophan, and L-isoleucine) correlated with enhanced immune function, whereas four metabolites (including L-glutamine, phosphoenolpyruvate, ribose 5-phosphate, and phosphoribosyl pyrophosphate) showed marked reduction, suggesting suppressed phosphorylation processes that mitigate alcohol-induced hepatotoxicity. In glycerophospholipid metabolism, nine differential metabolites were identified, with up-regulation of L-serine and α-acylglycerophosphocholine contrasting with down-regulation of seven metabolites (e.g., phosphatidylcholine, glycerophosphocholine, and citicoline). The observed alterations in choline metabolism (a key component of glycerophospholipid metabolism) further substantiate VVFP’s regulatory effects on these interconnected pathways. Our findings demonstrate that VVFP alleviates alcohol-induced liver injury through coordinated modulation of amino acid biosynthesis and glycerophospholipid metabolism. This mechanistic alignment is consistent with prior studies showing that glycerophospholipid remodeling mitigates ethanol hepatotoxicity [23,24,25]. Additionally, recent evidence indicates that amino acid metabolism counteracts alcohol-induced hepatic damage via energy homeostasis regulation [26,27], a pathway corroborated by the metabolic interactions identified in our study.

### 3.5. Combined Transcriptome and Metabolomics Analysis

To delineate gene–metabolite interplay, an integrated analysis of transcriptomic and metabolomic profiles was conducted between MK and M groups. Quantification of shared/unique KEGG pathways revealed 111 significantly enriched pathways, with 188 transcript-specific (58.4%) and 23 metabolite-specific (7.1%) pathways.

**Figure 7:**
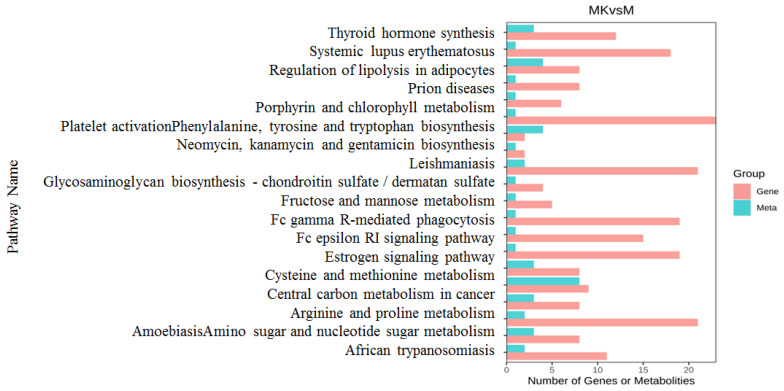
KEGG pathway enrichment of differential genes and metabolites.

For functional prioritization, the top 20 pathways ranked by *p*-value were visualized in a dual-axis bar plot (Figure 7), highlighting pathway-specific enrichment patterns of differentially expressed genes (DEGs) and differential metabolites (DMs). Differentially expressed genes were predominantly abundant in the metabolism of arginine and proline; related metabolic pathways, such as the metabolism of cysteine and methionine and central carbon metabolism in cancer; and human illnesses, such as systemic lupus erythematosus, amoebiasis, and leishmaniasis.

**Figure 8:**
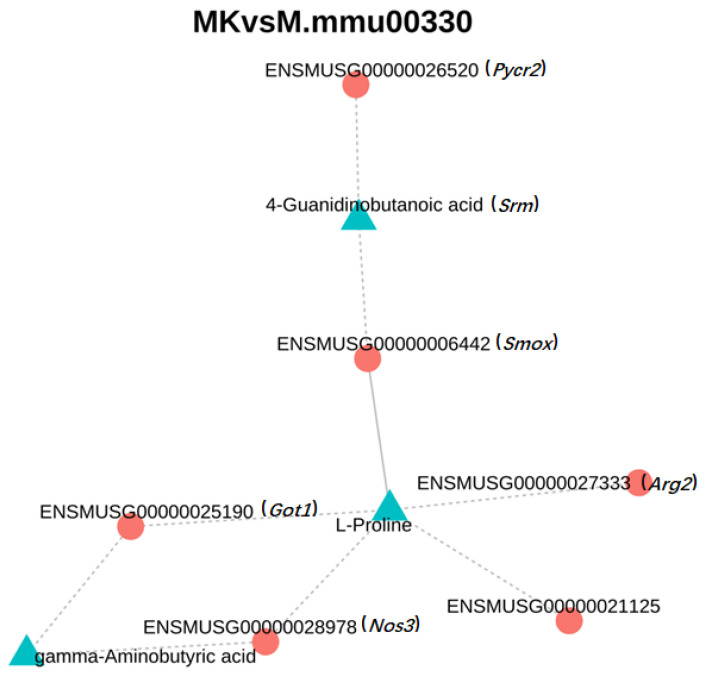
Arginine and proline metabolism differential genes and metabolite correlation network diagram. Note: *Pycr2*: ENSMUSG00000026520, Pyrroline-5-carboxylate reductase-like 2; *Srm*: ENSMUSG00000006442, Spermidine synthase; *Smox*: ENSMUSG00000027333, Spermine oxidase; *Arg2*: ENSMUSG00000021125, Arginase; *Got1*: ENSMUSG00000025190, Glutamate oxaloacetate transaminase 1; *Nos3*: ENSMUSG00000028978, Nitric oxide synthase.

To further elucidate the protective mechanism of VVFP against acute alcoholic hepatitis in mice, we identified arginine and proline metabolism as the most prominently co-enriched pathways. Within this pathway, three differentially abundant metabolites and eight differentially expressed genes were detected. To investigate the regulatory interplay, a correlation network (Figure 8) was constructed using the screened metabolites and genes (correlation coefficient ≥ 0.8).

The bipartite network depicted genes in red and metabolites in blue, and solid/dashed lines indicate positive/negative correlations. Key findings revealed the following: the metabolite 4-guanidinobutyric acid negatively correlated with *Pycr2* (ENSMUSG00000026520) and *Srm* (ENSMUSG00000006442), while L-proline positively correlated with *Srm* and negatively with *Got1* (ENSMUSG00000025190), *Nos3* (ENSMUSG00000028978), *Arg2* (ENSMUSG00000021125), and *Smox* (ENSMUSG00000027333). Additionally, γ-aminobutyric acid (GABA) showed negative correlations with *Got1* and *Nos3*. These hub genes *Srm* (spermidine synthase, polyamine-mediated cytoprotection), *Got1* (glutamate oxaloacetate transaminase, nitrogen balance), and *Arg2* (mitochondrial arginase, redox signaling modulation) collectively orchestrate metabolic adaptations that counteract alcohol-induced hepatic dysfunction. These interactions, particularly with metabolites such as L-proline and GABA, underscore VVFP’s therapeutic efficacy in alleviating oxidative stress, enhancing ammonia detoxification, and restoring redox equilibrium via targeted regulation of arginine–proline metabolism pathways.

## 4. Discussion

Transcriptome sequencing, as an advanced gene detection technology, enables the comprehensive and rapid acquisition of transcript sequence information from specific tissues through high-throughput sequencing and has been widely applied across multiple research domains [28]. In our investigation, deep transcriptome sequencing was performed on mouse liver tissues. The GO functional annotation and enrichment analysis revealed that differentially expressed genes were predominantly associated with stress responses and immune regulation. Notably, oxidative stress emerged as a central feature in acute alcohol-induced liver injury, serving as a key etiological mechanism—a finding consistent with previous studies. For instance, Xu et al. demonstrated that evening primrose B ameliorates alcoholic liver disease in mice through oxidative stress mitigation and intestinal flora modulation [29]. Similarly, Pan et al. [30] investigated ginsenoside Rc’s effects on alcohol metabolism, revealing its hepatoprotective role via SIRT6/NRF2 pathway activation to counteract oxidative stress in alcohol-related liver disease (ALD). Immune regulation, a fundamental physiological process maintaining dynamic homeostasis and systemic stability, becomes compromised during alcohol abuse, predisposing organisms to various pathologies [31]. Supporting this, Li et al. reported berberine’s therapeutic potential in alcohol-induced liver injury through activation of hepatic immunosuppressive cell populations [32]. This aligns with the established understanding that excessive alcohol consumption impairs cellular immunity. Complementary research by Wang et al. evaluated acetic acid bacteria’s immunomodulatory properties, suggesting their potential in mitigating alcohol-induced hepatotoxicity [33].

Our KEGG database enrichment analysis suggests that VVFP may exert protective effects through endoplasmic reticulum (ER) protein processing pathways. As the primary site for biosynthesis of proteins, lipids, and glycans, the ER coordinates complex post-translational modifications [34]. Notably, nearly all ER-synthesized proteins undergo glycosylation—an enzymatic process involving precise addition/removal of sugar moieties [35]. Given that advanced glycation end products (AGEs), primarily metabolized in the liver [36], accumulate during alcohol-induced hepatorenal dysfunction, their pathogenic role in hepatic deterioration becomes evident. We hypothesize that VVFP attenuates AGE formation by modulating ER protein processing pathways, thereby providing hepatoprotection.

Metabonomics serves as a critical interface bridging genetic expression and tissue function, facilitating biological information transfer [37]. This analytical approach systematically examines metabolic pathways of endogenous and exogenous metabolites, providing holistic insights into systemic pathological states. In our experimental design, UHPLC-MS/MS technology was employed to investigate metabolic alterations in murine models of acute alcoholic liver injury treated with VVFP. Comparative analysis of hepatic metabolites across treatment groups identified lipids/lipid-like molecules and organic acids/derivatives as predominant metabolic mediators. While lipids constitute essential nutrients supplying energy and fatty acids, their excessive accumulation predisposes organisms to metabolic disorders. Notably, chronic alcohol consumption induces lipid peroxidation, generating reactive acetaldehyde that accumulates due to impaired conversion to acetate, thereby disrupting redox homeostasis and exacerbating hepatic oxidative stress—a recognized precursor to fatty liver and alcoholic hepatitis [38]. Organic acids (excluding amino acids) demonstrate distinct hepatoprotective properties. Specifically, aromatic organic acids exhibit therapeutic potential against alcohol-induced hepatitis, as evidenced by Pan et al. [39], who documented Prunus mume-derived aromatic acids suppressing hepatocyte apoptosis via ROS-mediated p53 pathway modulation in murine models.

Our KEGG enrichment analysis highlighted amino acid biosynthesis and glycerophospholipid metabolism as pivotal pathways mediating VVFP’s hepatoprotection. Amino acids (including glutamate, arginine, and aspartate) represent fundamental protein constituents with emerging therapeutic applications in hepatic disorders. Concurrently, glycerophospholipids—the predominant phospholipid class—modulate alcohol-induced hepatocyte apoptosis [25]. Supporting findings include Cao et al. [23], who demonstrated Tartary buckwheat extract’s capacity to regulate phosphatidylcholine/phosphatidylethreonine ratios for ALD prevention, and Wu et al. [24], who elucidated leonurine hydrochloride’s mechanism through glycerophospholipid metabolic engagement—observations aligning with our conclusions.

Integrated transcriptomic–metabonomic analysis revealed VVFP’s modulation of arginine/proline metabolism, affecting eight target genes (e.g., *Got1*, *Arg2*, *Srm*) and differential metabolites (including γ-aminobutyric acid (GABA), L-proline, and 4-guanylbutyric acid). Of particular significance, GABA—a multifunctional non-protein amino acid—exerts hepatorenal protection and metabolic disorder mitigation [40]. Mechanistically, Wang et al. [41] established GABA’s anti-apoptotic effects via IRE1α-ASK1-JNK pathway inhibition, while Park et al. [42] attributed fermented daylily extract’s hepatoprotection to GABA-mediated antioxidant enhancement and lipid metabolism regulation. These collective findings substantiate VVFP’s therapeutic efficacy against alcohol-induced hepatotoxicity. While this study provides valuable insights, several limitations should be acknowledged. These include reliance on short-term experimental models, absence of validation in human cell systems, and insufficient integration of gut microbiota analysis. Future research will prioritize functional validation of VVFP in human-relevant models, long-term intervention studies, and exploration of its interactions with gut microbiota to comprehensively assess therapeutic potential.

## 5. Conclusions

**Figure 9:**
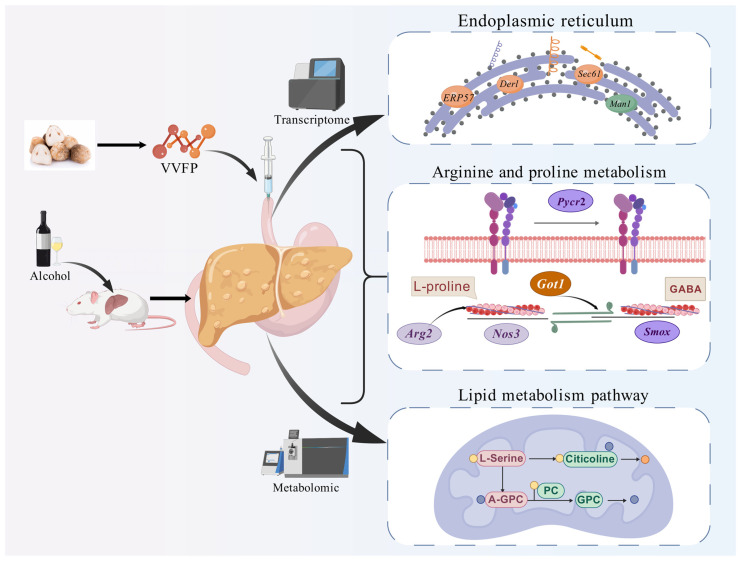
The hepatoprotective mechanisms of VVFP against acute alcohol-induced liver injury in murine models. Note: *ERP57*: ENSMUSG00000027248, Endoplasmic reticulum–resident protein 57; *Derl*: ENSMUSG00000075701, Derlin protein; *Sec61*: ENSMUSG00000030082, Translocon alpha subunit Sec61; *Man1*: ENSMUSG00000037306, Mannosidase alpha-like 1; *Pycr2*: ENSMUSG00000026520, Pyrroline-5-carboxylate reductase-like 2; *Arg2*: ENSMUSG00000021125, Arginase; *Nos3*: ENSMUSG00000028978, Nitric oxide synthase; *Got1*: ENSMUSG00000025190, Glutamate oxaloacetate transaminase 1; *Smox*: ENSMUSG00000027333, Spermine oxidase.

This multi-omics study demonstrates that *V. volvacea* polypeptide (VVFP) alleviates acute alcohol-induced liver injury through coordinated molecular and metabolic reprogramming. VVFP pretreatment significantly prolonged ethanol tolerance latency (7.43-fold at 200 mg/kg, *p* < 0.01) and accelerated sobriety recovery (65.1% reduction vs. model), while reducing hepatic edema (liver index restored to near-physiological levels, 4.71% vs. 5.66% in model). Transcriptomics revealed VVFP’s suppression of endoplasmic reticulum stress via up-regulation of 36 genes (e.g., *ERP57*, *Sec61*, *Derl*) in the protein processing pathway (KEGG:04141), mitigating alcohol-induced proteotoxic damage. Metabolomics identified restoration of phosphatidylcholine homeostasis (16:0/18:1-PC increased 4.8-fold) and organic acid flux through glycerophospholipid and amino acid metabolism. Integrated analysis uncovered a core regulatory network linking *Got1*, *Arg2*, and *Srm* with metabolites (4-guanidinobutyric acid, GABA, L-proline) via linoleic acid metabolism, synergistically addressing hepatic dysfunction and oxidative stress (Figure 9). These findings establish VVFP as a multi-target hepatoprotective agent, providing mechanistic insights and actionable biomarkers (e.g., *Got1*-GABA axis) for developing functional foods against alcohol-related liver disease.

## Figures and Tables

**Table 1:** Dose–response effects of VVFP on ethanol metabolism parameters.

Treatment	Dose (mg/kg)	Intoxication Latency (min)	Sobriety Recovery (min)
Control	-	0.00 ± 0.00	0.00 ± 0.00
Model	-	9.333 ± 0.471 **	216.667 ± 3.859 **
Positive	200	32.667 ± 1.700 **^##^	96.667 ± 4.110 **^##^
L-VVFP	100	23.667 ± 1.247 **^##^	139.667 ± 3.300 **^##^
M-VVFP	200	69.333 ± 2.625 **^##^	75.667 ± 4.190 **^##^
H-VVFP	400	51.333 ± 0.943 **^##^	88.333 ± 0.471 **^##^

Note: Data expressed as mean ± SEM (n = 12). Statistical significance: ** *p* < 0.01 vs. Control; ^##^ *p* < 0.01 vs. Model (Two-way ANOVA with Tukey’s multiple comparisons).

**Table 2:** Dose-dependent amelioration of alcohol-induced hepatomegaly by VVFP.

Treatment	Dose (mg/kg)	Liver Weight (g)	Mouse Weight (g)	Liver Index (%)
Control	-	1.573 ± 0.021	35.077 ± 0.319	4.485 ± 0.034
Model	-	1.840 ± 0.041 **	32.565 ± 0.272 **	5.663 ± 0.088 **
Positive	200	1.537 ± 0.009 **^##^	34.023 ± 0.411 *^#^	4.535 ± 0.054 ^##^
L-VVFP	100	1.613 ± 0.005 **^##^	33.448 ± 0.361 **^#^	4.825 ± 0.057 **^##^
M-VVFP	200	1.593 ± 0.012 **^##^	33.794 ± 0.482 *^#^	4.707 ± 0.092 *^##^
H-VVFP	400	1.583 ± 0.021 *^##^	33.690 ± 0.436 *^#^	4.700 ± 0.075 *^##^

Note: Data expressed as mean ± SEM (n = 12). Statistical significance: * *p* < 0.05 vs. Control; ** *p* < 0.01 vs. Control; ^#^ *p* < 0.05 vs. Model; ^##^ *p* < 0.01 vs. Model (Two-way ANOVA with Tukey’s post hoc test).

## Data Availability

The original contributions presented in the study are included in the article, further inquiries can be directed to the corresponding authors.
